# The Effectiveness and Safety of Tranexamic Acid in Treating Aneurysmal Subarachnoid Hemorrhage: A Systematic Review and Meta-Analysis

**DOI:** 10.3390/healthcare12232452

**Published:** 2024-12-05

**Authors:** Mohammed O. Al Zayer, Fatima M. Al Johani, Shahad A. Al ghamdi, Mohammed D. Al Hejaili, Fatima H. Al Mukhtar, Arwa M. Al Ariany, Bashar H. Al Anazi, Khalid A. Al Mutairi, Rammaz H. Khoja, Haidar F. Al Amer, Adel A. Zeidan, Dunya Al Faraj

**Affiliations:** 1 College of Medicine, Imam Abdulrahman Bin Faisal University, Dammam 34212, Eastern Province, Saudi Arabia; dr.fatimhussain2022@gmail.com (F.H.A.M.); zeidan.adel01@gmail.com (A.A.Z.); 2 Department of Emergency, King Fahad Hospital of the University, Al-Khobar 34445, Eastern Province, Saudi Arabia; dr.alamerh@gmail.com (H.F.A.A.); dnfaraj@iau.edu.sa (D.A.F.); 3 Department of Emergency, King Salman Bin Abdulaziz Medical City, Al-Madinah 42319, Al-Madinah Province, Saudi Arabia; fatimaaljohani8@gmail.com; 4 College of Pharmacy, King Abdulaziz University, Jeddah 21589, Makkah Province, Saudi Arabia; shahadgh20@gmail.com; 5 College of Medicine, Taibah University, Al-Madinah 42353, Al-Madinah Province, Saudi Arabia; mohammed.alhejaili30@gmail.com (M.D.A.H.); remaz35040234@gmail.com (R.H.K.); 6 College of Medicine, Jeddah University, Jeddah 23218, Makkah Province, Saudi Arabia; arwa.alariany1@gmail.com; 7 Department of Emergency, Prince Sultan Military Medical City, Riyadh 12233, Al-Riyadh Province, Saudi Arabia; bashaaralenzy@gmail.com; 8 College of Medicine, Shaqra University, Shaqra 15526, Al-Riyadh Province, Saudi Arabia; khalid.iu06@gmail.com

**Keywords:** tranexamic acid, subarachnoid hemorrhage, efficacy, safety, rebleeding, randomized controlled trials

## Abstract

**Background/Objectives:** Tranexamic acid (TXA) is a synthetic antifibrinolytic agent that inhibits plasminogen activation, thereby reducing bleeding. The aim of this systematic review was to investigate its role in aneurysmal subarachnoid hemorrhage (SAH)—a condition indicated by bleeding between two layers of brain tissue—to stop rebleeding and improve patient outcomes. **Methods:** We conducted a systematic review and meta-analysis of randomized controlled trials from 1981 to 2024, focusing on the efficacy and safety of TXA in treating aneurysmal SAH (PROSPERO registration: CRD42024504834). Our comprehensive search of the PubMed and Cochrane Library databases identified studies assessing TXA at dosages of 3 to 6 g per day and examining outcomes such as rebleeding incidence, mortality, thromboembolic events, and other adverse effects. **Results:** From six included studies involving 2990 patients, the meta-analysis showed TXA largely lowered rebleeding risk (OR 0.54 95% CI 0.43–0.68; *p* < 0.00001), yet mortality rates were not largely different between the TXA group (385 out of 1201), and the control group (344 out of 1193) (OR 1.18 95% CI 0.98–1.40; *p* = 0.07). Likewise, there were no large differences in the occurrence of cerebral ischemia and blood clot-related events between the groups. **Conclusions:** TXA effectively reduces the risk of rebleeding in SAH patients, but does not significantly alter mortality or the incidence of thromboembolic complications. These findings back the careful use of TXA and demonstrate the need for further research to better its clinical use and assess long-term impacts.

## 1. Introduction

Globally, almost 9 out of 100,000 people encounter subarachnoid hemorrhage (SAH) [1], which is bleeding within the subarachnoid space underneath the cerebral arachnoid membrane due to a head injury or blood vessels rupturing spontaneously. Spontaneous blood vessel rupturing has been categorized into three causes: aneurysmal, non-aneurysmal, and perimesencephalic. Ruptured intracranial aneurysms—protrusions of cerebral blood vessels—are the most common cause of SAH [2]. Elevated blood pressure, smoking, and a family history of cerebral aneurysms are significant predictors of brain aneurysms.

Potential causes of ruptured aneurysms include aneurysm site and extent, hypertension, smoking, cocaine misuse, female gender, and advanced age. They may manifest as severe headaches, disorientation, hemiparesis, focal cerebral impairments, or even coma [3]. Aneurysmal subarachnoid hemorrhage (aSAH) has a mortality rate of 30% in 40% of cases due to secondary bleeding of the aneurysm [4]. Alongside the high mortality rate, SAH has been associated with poor clinical outcomes. This leaves patients to experience long-term impairments in memory, planning, problem-solving, decision-making, speech, and activities of daily living (ADLs) such as eating, toileting, dressing, and showering. They may also struggle with instrumental activities of daily living (IADLs), such as handling money, shopping, and routine maintenance, and may suffer from depression [5].

Given the severe implications and high mortality, managing SAH should be a top priority in emergency departments. According to recent management guidelines for aSAH, early surgical intervention within 72 h, or optimally within 48 h, is associated with a reduction in the risk of rebleeding, which is highest in the first 24 h [6,7]. However, rebleeding may occur due to unavoidable delays in surgical intervention, potentially preventable by administering tranexamic acid (TXA) [8].

Tranexamic acid (TXA) acts as a lysine analogue, binding to plasminogen to inhibit fibrinolysis and thereby reduce bleeding. Since its introduction to the market, TXA has been commonly used to reduce blood loss from surgery, severe trauma, and heavy menstruation [8]. Several studies have investigated the effects of TXA in cases of ruptured aneurysmal SAH. While most studies have shown that TXA can decrease rebleeding [7,8,9], they have not provided evidence of its effectiveness in improving poor outcomes or reducing mortality [10]. The potential rise in the risk of cerebral ischemia may counterbalance the impact of TXA on rebleeding [8]. Although there have been reviews on the efficacy of TXA in managing SAH concluding that it cannot be routinely used, these reviews acknowledged certain limitations in their methodology, included low-quality studies, and encouraged a need for further assessment of the use of TXA on patients with SAH [11].

The role of TXA in managing SAH is an ongoing investigation. This systematic review aims to synthesize the existing body of evidence on the effectiveness and safety of tranexamic acid as an adjunctive therapy in treating subarachnoid hemorrhage. By comprehensively analyzing randomized controlled trials (RCTs), we seek to elucidate the potential benefits and risks of using TXA in this clinical context.

## 2. Materials and Methods

### 2.1. Literature and Search Strategy

This systematic review and meta-analysis was conducted in strict accordance with the Preferred Reporting Items for Systematic Reviews and Meta-Analyses (PRISMA) 2020 guidelines. All relevant PRISMA checklist items, including structured reporting of objectives, eligibility criteria, search strategy, study selection, data extraction, risk-of-bias assessment, and synthesis of results, have been followed throughout the manuscript. A completed PRISMA checklist is included as Supplementary Material to ensure transparency and adherence to these guidelines. Additionally, the study was prospectively registered in the PROSPERO database (CRD42024504834), with the protocol accessible through the PROSPERO website. The study aimed to evaluate the clinical efficacy and safety of tranexamic acid (TXA) in treating aneurysmal subarachnoid hemorrhage (aSAH). A comprehensive electronic search of the PubMed and Cochrane Library databases was performed in January 2024, covering publications from 1981 to 2024. The search strategy, independently designed by two authors (MZ, FJ) and approved by the rest of the team, included keywords such as “efficacy of tranexamic acid”, “tranexamic acid”, “aneurysmal subarachnoid hemorrhage”, “randomized controlled trials”, “SAH”, and “TXA”.

The relevant studies targeted a population of patients diagnosed with aneurysmal subarachnoid hemorrhage (aSAH) who received tranexamic acid (TXA) compared to control groups treated with a placebo. This systematic review aims to assess the outcomes of TXA use in SAH, focusing on the incidence of rebleeding, mortality rate, thromboembolic events, and cerebral ischemia.

### 2.2. Inclusion and Exclusion Criteria

This review included all clinical studies published between 1981 and 2024 that reported on the use of tranexamic acid (TXA) to treat aneurysmal subarachnoid hemorrhage (SAH). Only studies published in peer-reviewed journals and in the English language were considered, focusing solely on randomized controlled trials, to avoid translation inaccuracies, minimize bias, and provide stronger evidence of causality. The studies included varied dosages of TXA, ranging from 3 to 6 g per day, and reported on relevant clinical outcomes such as the incidence of rebleeding, mortality, and other significant outcomes associated with TXA use.

The exclusion criteria were stringent, filtering out studies that did not report the outcomes of interest, did not focus on patients with aneurysmal SAH, or did not use TXA to treat SAH. Additionally, non-randomized controlled trials, including observational studies, case reports, narrative reviews, editorials, and letters, were excluded. Studies assessed to have a high risk of bias or low quality based on study design, sample size, data collection, or analysis were also excluded. Furthermore, full-text studies not published in English or not available in the searched databases were omitted from this review.

### 2.3. Data Extraction

The data extracted from the relevant studies were meticulously categorized into four major areas: study characteristics, patient characteristics, intervention characteristics, and outcome measures.

For study characteristics, we gathered information such as author, year of publication, country, study design, sample size, follow-up duration, severity of aSAH, and inclusion/exclusion criteria.

Patient characteristics included age, sex, medical history, medications, preoperative hemoglobin level, and any bleeding disorders.

Intervention characteristics covered the dosage of tranexamic acid, timing of administration, and mode of delivery.

Additionally, we extracted outcome measures that were crucial for assessing the efficacy and safety of TXA. These included the incidence of rebleeding, mortality rate, thromboembolic events, adverse effects, length of hospital stay, and quality of life. For data analysis, we employed robust statistical methods, including calculations of effect size, confidence intervals, and assessments of heterogeneity and publication bias. This comprehensive approach ensured a thorough evaluation of the clinical efficacy and safety of TXA in treating aneurysmal subarachnoid hemorrhage.

### 2.4. Risk-of-Bias Assessment

The quality of RCTs was assessed using the Cochrane risk-of-bias tool. The judgments within each tool’s several domains were combined to provide an overall RoB2 judgment that included five major domains. These domains are set in stone, concentrating on trial design, conduct, and reporting elements while employing “signaling questions” to extract pertinent data regarding bias risk.

This is then evaluated using an algorithm, and the results can be classified as “low” (meaning that there is little risk of bias across all domains), “some concerns” (meaning that there is some concern across at least one domain), or “high” (meaning that there is a high risk across many domains or that there are some concerns across multiple domains). The risk-of-bias assessment was conducted independently by two authors (BA, KM), and disagreements were resolved with consensus after consultation with senior authors. While a formal assessment of the certainty of evidence was not conducted, the quality of the included studies was thoroughly evaluated through a detailed risk of bias assessment using the Cochrane risk-of-bias tool. The results are interpreted considering this quality assessment, ensuring that the conclusions drawn are based on the rigor of the available evidence.

### 2.5. Statistical Meta-Analysis

For statistical analysis and reference management of the extracted studies, the Review Manager 5.4 program (Cochrane Collaboration, Oxford, UK, 2020) was used. Forest plots were then used to compare the outcomes of rebleeding, mortality rate, thromboembolic events, and cerebral ischemia in the tranexamic treatment group and placebo control group.

The summary statistics used a confidence interval (CI) of 95% to pool the different outcome variables as well as pooled odd ratios (ORs) for dichotomous outcomes. In order to overcome heterogeneity, the I^2^, X^2^, and *p*-values were utilized. Heterogeneity was defined as *p* < 0.01, and an I^2^ of 0% to 25% was deemed to have low statistical heterogeneity, an I^2^ of 25% to 75% was deemed to have moderate heterogeneity, and an I^2^ of more than 75% was considered to have high heterogeneity. A fixed-effect model was used.

## 3. Results

### 3.1. Selection Process

The search began with a robust collection of 1095 articles from PubMed and the Cochrane Library. After meticulously reviewing titles and abstracts, we selected 13 studies for in-depth evaluation. To ensure no relevant research was overlooked, we leveraged the Rayyan platform to examine references from these studies and uncover additional pertinent articles.

**Figure 1:**
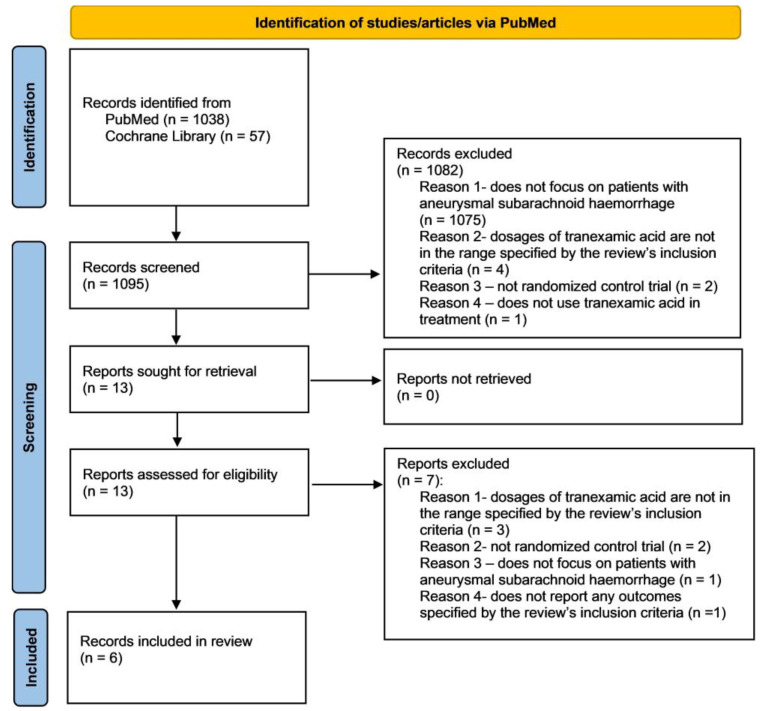
Flow diagram of the search strategy and study selection process for this systematic review with meta-analysis.

From this refined set, we excluded three studies due to tranexamic acid dosages not within this review’s inclusion criteria, two that lacked the randomized controlled trial design, one that did not focus on aneurysmal subarachnoid hemorrhage, and one that failed to address any crucial clinical outcomes stated in this review’s inclusion criteria. After removing duplicates, our final analysis included six high-quality studies, as represented by the PRISMA flow diagram in Figure 1.

### 3.2. Characteristics of Included Studies

The characteristics of the studies included in the trials are shown in Table 1. A total of six studies were included, half conducted in Sweden and the other half in Amsterdam. The total number of participants varied from one study to another. However, the total participants included in the six analyzed studies was 2990. As for the distribution of gender differences between trials, most of the studies had a balanced number between males and females. For age groups, one trial studied age as a subcategory and the most frequent age group was from 50 to 69 years old, while for the other studies, the mean age ranged in the 50s with an overall mean age of approximately 55.06 years.

The clinical characteristics between the different study groups in the included studies are compared in Table 2. None of the included studies examined the participants’ medical history, except for a study that found 19 patients with arterial hypertension and another study that found around 20% of each group had been diagnosed with hypertension. For medications, most of the participants received bedrest and sedation whether they were in the treatment group or control group. Other medications, such as anti-hypertensives, anti-coagulants, platelet inhibitors, anti-epileptic drugs, corticosteroids, and nimodipine, were used in some studies. Another study’s participants received nimodipine. Regarding the duration of the treatment, some did not exceed 48–72 h, but others continued for 3–6 weeks. The daily dosages ranged from 1 g to 6 g. The initial dose was administered within 24–72 h. Intravenous delivery was used in all of the studies, except for three studies where the intravenous route was initially used and then administration continued orally.

### 3.3. Outcomes of Tranexamic Acid Use

The differences in adverse events (rebleeding, mortality rate, thromboembolic events, cerebral ischemia, and other adverse effects) between the treatment group and control group for all included studies are shown in Table 3. The incidence of rebleeding varied among studies, with rates ranging from 6 to 42 cases in the treatment groups and 7 to 57 cases in the control groups. Death was also common, as the mortality rate ranged from 13 to 128 cases in the TXA treatment groups and 9 to 120 cases in the control groups. However, thromboembolic events were less common, as the incidence of such events was 4 to 34 cases in the treatment groups and 3 to 40 cases in the control groups. Cerebral ischemia occurred in both groups. In the treatment groups, 5 to 108 cases suffered from cerebral ischemia across the studies, and 2 to 106 cases had cerebral ischemia in the control groups. Other adverse events were described in only three studies: 46 cases of ventricular dilatation, 8 cases of myocardial infarctions or suspected infarctions, 33 cases of vasospasms, 20 cases of clinical deteriorations from onset, 133 cases of hydrocephalus, 32 cases of post-operative ischemia, and 58 cases of other adverse effects.

**Figure 2:**
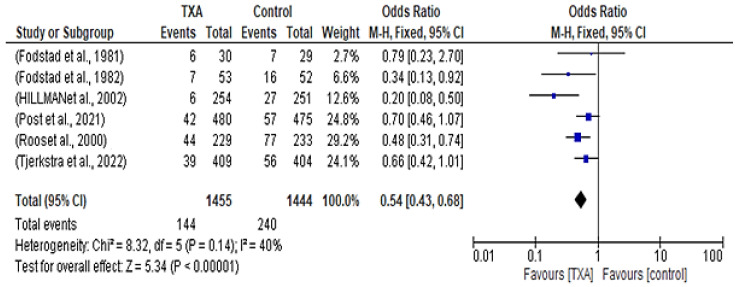
Forest plot comparing tranexamic acid and control groups for incidence of rebleeding [10,12,13,14,15,16].

The study analyzed data from six interveinal studies comparing rebleeding incidence between patients who received TXA versus control groups. Of 1445 patients in the treatment group, 144 of them had rebleeding. On the other hand, 240 out of 1444 patients in the control group suffered from rebleeding. The fixed-effect meta-analysis indicated a higher risk of rebleeding in the control group compared to the TXA group, with an odds ratio of 0.54 (95% CI 0.43–0.68; *p* < 0.00001) and with moderate statistical heterogeneity (χ2 = 8.32; I2 = 40%; *p* = 0.14) observed, as shown in Figure 2.

**Figure 3:**
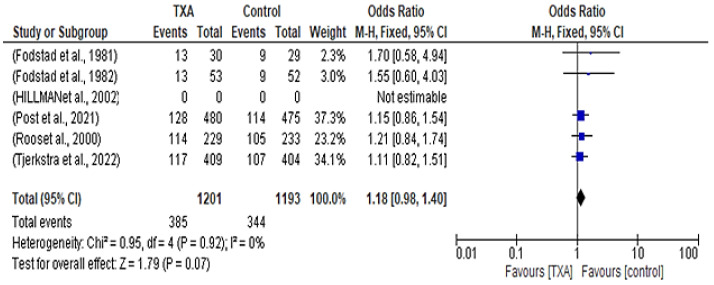
Forest plot comparing tranexamic acid and control groups for mortality rate [10,12,13,14,15,16].

Regarding the mortality rate between patients who received TXA versus controls, only five studies contributed data. Among 1201 patients in the treatment group, 385 of them died. In contrast, 344 out of the 1193 patients in the control group died. The forest plot indicates no significant difference in mortality rates between the control group and the TXA group, with an odds ratio of 1.18 (95% CI 0.98–1.40; *p* = 0.07) and with no heterogeneity observed, as shown in Figure 3.

**Figure 4:**
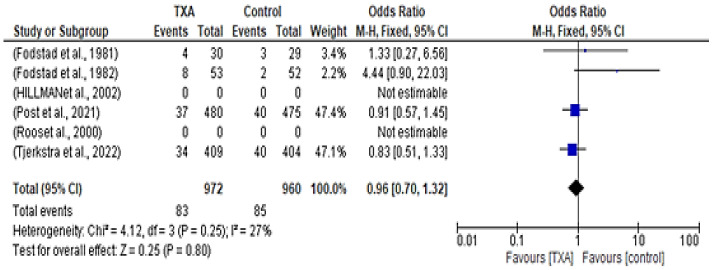
Forest plot comparing tranexamic acid and control groups for thromboembolic events [10,12,13,14,15,16].

Only four out of the six included studies discussed thromboembolic events, in which 83 out of 972 patients in the treatment group experienced thromboembolic events, while 85 out of 960 patients in the control group experienced a similar event. Analysis showed that there was no significant difference in thromboembolic events between the groups, with an OR of 0.96 (95% CI 0.70–1.32; *p* = 0.80) and 27% heterogeneity observed (χ^2^= 4.13, *p* = 0.25), as shown in Figure 4.

**Figure 5:**
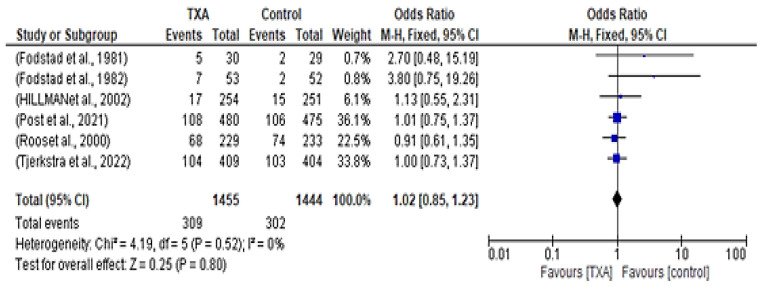
Forest plot comparing tranexamic acid and control groups for cerebral ischemia [10,12,13,14,15,16].

Regarding the occurrence of cerebral ischemia, all of the six included studies examined this event. In the treatment group, 309 out of the 1455 patients had cerebral ischemia. On the other hand, 302 out of the 1444 patients in the control had cerebral ischemia. The forest plot in Figure 5 shows that there was no significant difference in the occurrence of cerebral ischemia between the two groups, with an OR of 1.02 (95% CI 0.85–1.23; *p* = 0.80) and no heterogeneity observed.

### 3.4. Risk-of-Bias Assessment of the Included Studies

**Figure 6:**
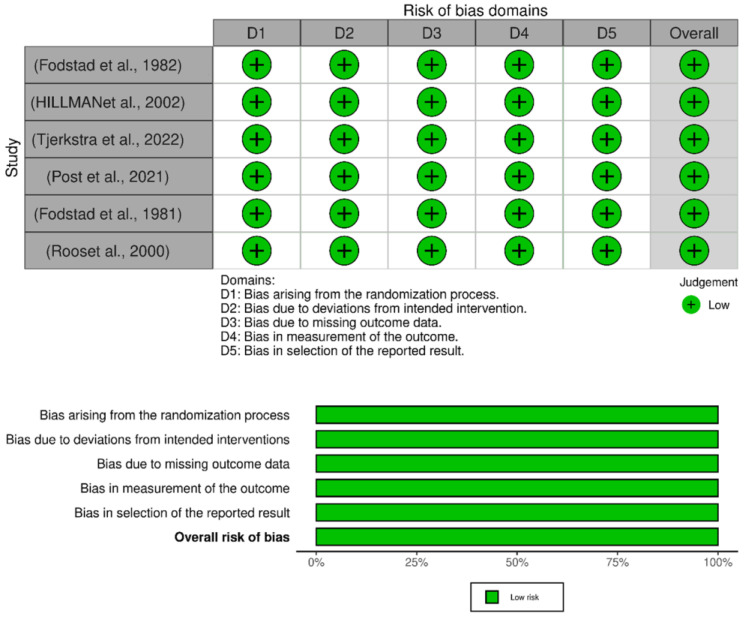
Risk of bias assessment of the included articles [10,12,13,14,15,16].

The included studies were assessed for risk of bias using five domains. All six studies scored low on all the domains of the risk-of-bias tool, as shown in Figure 6.

## 4. Discussion

Subarachnoid hemorrhage (SAH) occurs when there is bleeding in the space between the arachnoid membrane and the pia mater, which is called the subarachnoid space. It is classified as traumatic SAH (tSAH) or spontaneous SAH (sSAH). Most spontaneous (non-traumatic) SAH instances result from the rupture of an intracranial aneurysm or aneurysmal SAH (aSAH) [17]. However, around 20% of SAH cases occur without an aneurysm rupture. Although there are various potential causes for non-aneurysmal SAH (naSAH), the source of bleeding occasionally remains unidentified [18]. Thus, the focus of this review was to evaluate patients diagnosed with aSAH instead of other types of bleeding in the brain to strengthen the reliability and generalizability of the findings.

Tranexamic acid (TXA) acts as an antifibrinolytic by blocking the transition of plasminogen to plasmin [19]. TXA has been widely used to control blood loss during surgeries, severe traumatic injuries, and heavy menstruation. Researchers have also investigated its role in managing SAH [11,20]. Although the American Heart Association–American Stroke Association 2023 guideline for the management of patients with aneurysmal subarachnoid hemorrhage stated, with a strong level of evidence, that the routine use of anti-fibrinolytic therapy has no benefit in improving functional outcomes, it also stated that the use of TXA as a short-term therapy in cases where aneurysm treatment is delayed is a possibility. Moreover, it stated that there are knowledge gaps in terms of antifibrinolytic therapy and that this is a target for future research [6]. The chemical formulation of TXA has been consistent throughout its introduction in the 1960s. Although TXA has been on the market since the 1960s, clinical trials to investigate its use in SAH only started in the late 1970s and early 1980s. This supports our timeframe selection starting from 1981 and ensures that the same formulation has been administered in the included studies. Various studies concluded that TXA should be administered at 10 mg/kg intravenously every 6–8 h for local fibrinolytic bleeding, 10 mg/kg intravenously every 3–4 h for systemic fibrinolytic bleeding, and 10–20 mg/kg every 6–8 h if given orally. This systematic review included studies that administered dosages of 3–6 g per day to capture the common clinical practices and provide a balanced view of the outcomes across different acceptable dosages. Additionally, it allows the review to account for dose–response effects and enables a better understanding of how varying doses can impact clinical outcomes (8).

### 4.1. Main Findings and Implications

Our systematic review, which analyzed data from six studies conducted in Sweden and Amsterdam, including 2990 patients, aimed to assess the efficacy and safety of tranexamic acid (TXA) in treating aneurysmal subarachnoid hemorrhage (aSAH). The focus was on improving patient outcomes while minimizing complications. The findings revealed that TXA essentially reduced the risk of rebleeding among SAH patients, a promising result that could significantly improve patient outcomes. The odds ratio (OR) of 0.54 (95% CI 0.43–0.68) indicated an outstanding reduction in rebleeding risk for those treated with TXA (*p* < 0.00001) [21]. Moreover, the risk of bias across studies was assessed using a funnel plot, and no significant publication bias was detected. However, the possibility of reporting biases across studies cannot be completely ruled out.

Despite this benefit, TXA did not broadly impact mortality rates—385 out of 1201 patients in the TXA group died, while in the control group, 344 out of 1193 patients died (OR 1.18; 95% CI 0.98–1.40; *p* = 0.07). Similarly, occurrences of cerebral ischemia were similar between the TXA and control groups (OR 1.02; 95% CI 0.85–1.23; *p* = 0.80). Thromboembolic events were rare and equally distributed across groups (OR 0.96; 95% CI 0.70–1.32; *p* = 0.80).

These findings are consistent with some previous studies, although significant differences exist. A survey at Chattogram Maa-O-Shishu Hospital in July 2022 did not show the essential benefits of TXA in reducing rebleeding or improving one-month outcomes, though our review suggests significant rebleeding reductions with TXA [22]. This discrepancy emphasizes the variation in study designs and patient groups, showing the need for more consistent and rigorous research [18].

Our analysis shows TXA’s effectiveness in reducing rebleeding risk among SAH patients without predominantly affecting mortality or increasing the risks of thromboembolic events and cerebral ischemia [21,22]. This positions TXA as a valuable treatment in acute SAH management, particularly for rebleeding prevention. Nonetheless, the lack of a significant impact on mortality and other potential adverse effects, such as ventricular dilation and myocardial infarction (MI), requires clinicians to be selective and cautious in balancing the reduced rebleeding risk against potential adverse events on a case-by-case basis [22].

### 4.2. Limitations

Although it provides valuable insights, our review has limitations. Several studies had relatively small samples and short follow-up, which hampers the ability to detect all adverse effects or complications of TXA, especially in terms of long-term outcomes and quality-of-life measures. Future research should include larger samples and extended follow-up to ascertain TXA’s effectiveness and outcomes better. Reassessing TXA’s effects on SAH, identifying patient-specific factors that may influence treatment responses, and conducting longitudinal studies to examine long-term outcomes, quality of life, and cognitive/neuropsychological recovery for SAH survivors should become priorities [18,22]. This meta-analysis revealed moderate-to-high heterogeneity in certain outcomes, particularly in terms of rebleeding, which can affect the reliability of the pooled results. Although no significant publication bias was observed, only six studies were included in this review due to the strict inclusion criteria. This might influence the overall conclusions and prevent a thorough assessment of the effectiveness and safety of TXA in aSAH. Even though all included studies administered TXA within the acceptable standard range of dosing and duration, inconsistent treatment protocols in both TXA and control groups may introduce certain biases and confounding effects, which can complicate the generalizability of this review’s findings.

Our study indicates that TXA may reduce rebleeding risks in SAH patients, a significant finding that should reassure clinicians. However, it did not show significant results in terms of its impact on mortality rates or the risk of thromboembolic events and cerebral ischemia [20,21]. TXA may help prevent early rebleeding, but its overall effect on clinical outcomes remains uncertain. Additional research is necessary to optimize its use in SAH management, as these other outcomes are critical endpoints for clinical decision-making in SAH management [22]. The routine use of TXA for SAH should be considered only when more complete evidence becomes available [23].

### 4.3. Recommendations

Future research should aim to identify patient-specific factors that impact the response to TXA treatment and develop personalized management strategies. Longitudinal studies assessing TXA’s effects on long-term outcomes, quality of life, and cognitive recovery in SAH survivors are necessary to optimize its clinical use. Until more complete evidence is available, the benefits of reduced rebleeding against potential risks should guide the routine use of TXA in treating SAH.

## 5. Conclusions

In conclusion, according to our rigorous review and meta-analysis, tranexamic acid (TXA) has been shown to lower rebleeding risks broadly for aneurysmal subarachnoid hemorrhage (SAH) patients. The analysis reveals a significant reduction in the risk of rebleeding for those treated with TXA compared to control groups. However, TXA did not significantly affect overall mortality rates or the incidence of thromboembolic events and cerebral ischemia, suggesting uncertainty about its long-term impact on survival and other complications.

The findings showcase that TXA has the potential to be a beneficial adjunctive therapy in managing acute cases of SAH due to its ability to prevent rebleeding. Nevertheless, observed adverse effects such as ventricular dilatation and myocardial infarction show the need for careful patient selection and monitoring. The study emphasizes the need for more wide-ranging, rigorously designed clinical trials with larger samples and extended follow-up to fully elucidate TXA’s efficacy and safety profile.

## Figures and Tables

**Table 1:** Studies’ characteristics.

Author (Year)	Country	Journal Published in	Study Design	Number of Patients			Sex (Male: Female)			Age in Months (Mean)	
				TXA	Control	Total	TXA	Control	Total	TXA	Control
Fodstad et al. (1981) [12]	Sweden	Congress of Neurological Surgeons—Neurosurgery	Randomized controlled clinical trial	30	29	59	13:17	12:17	25:34	50	53
Fodstad et al. (1982) [13]	Sweden	Aeta Neurochirurgica	Randomized controlled clinical trial	53	52	105	13:17	12:17	25:34	50	53
Roos et al. (2000) [14]	Amsterdam	American Academy of Neurology—Neurology	Prospective, double-blind, placebo- controlled clinical trial	229	233	462	91:138	72:161	163:299	55	56
* Hillman et al. (2002) [15]	Sweden	Journal of Neurosurgery	Prospective randomized study	254	251	596	N/A	N/A	N/A	15–29 years (5), 30–49 years (72), 50–69 years (148), above 70 years (29)	15–29 years (5), 30–49 years (72), 50–69 years (148), above 70 years (29)
Post et al. (2021) [10]	Amsterdam	The Lancet	Prospective, randomized, controlled, open-label trial	480	475	955	148:332	163:312	311:644	58.4	58.4
Tjerkstra et al. (2022) [16]	Amsterdam	American Academy of Neurology—Neurology	Comparison study	409	404	813	112:297	123:281	235:578	58.4	58.4

* Hillman et al. (2002) [15]: age was taken in frequencies. N/A: not available, TXA: tranexamic acid.

**Table 2:** Comparing clinical characteristics between tranexamic acid groups and control groups.

Author (Year)	Medical History		Medications		Dosage. Timing, and Mode of Delivery of TXA
	TXA	Control	TXA	Control	
Fodstad et al. (1981) [12]	aSAH, 7 with arterial hypertension	aSAH, 12 with arterial hypertension	TXA with bedrest, sedation, and hypotensive drugs, anti-epileptic drugs, and corticosteroids	Bedrest, sedation, and hypotensive drugs, anti-epileptic drugs, and corticosteroids	First dose within 3 days. 1 g IV in 100 mL of saline every 4 h during 1st week and every 6 h during the 2nd week. During the 3rd through the 6th weeks, 1.5 g was given orally every 6 h until re-hemorrhage, operation, discharge, or death.
Fodstad et al. (1982) [13]	aSAH	aSAH	TXA with bedrest and sedation	Bedrest and sedation	First dose within 3 days. Series 1: 1 g IV six times daily for 1st week, 1 g IV four times daily from 2nd to 5th weeks, 1 g IV three times daily during 6th week until operation. Series 2: 1st and 2nd week similar to series 1, but 1.5 g orally 4 times daily from 3rd to 6th week until operation.
Roos et al. (2000) [14]	aSAH	aSAH	Both groups received nimodipine		First dose within 96 h. 1 g IV every 4 h during the 1st week, 1.5 g orally every 6 h in the 2nd and 3rd weeks for a maximum of 3 weeks until treatment of aneurysm, or adverse effects and complications arise.
Hillman et al. (2002) [15]	aSAH, hypertension (20.5%)	aSAH, hypertension (20.3%)	TXA	N/A	First dose within 48 h. 1 g IV at diagnosis, 1 g IV after 2 h, followed by 1g IV every 6 h until the aneurysm occluded or until 72 h after SAH. The mean dosage is 4.4 g.
Post et al. (2021) [10]	aSAH	aSAH	TXA, platelet inhibitor (16), anti-coagulant (15), anti-hypertensive (113)	Platelet inhibitor (16), anti-coagulant (19), anti-hypertensive (110)	First dose within 24 h. 1 g IV bolus at diagnosis, followed by continuous infusion of 1 g IV every 8 h until treatment of aneurysm or a maximum of 24 h.
Tjerkstra et al. (2022) [16]	aSAH	aSAH	TXA (393)	Usual care (402)	First dose within 24 h. 1 g IV bolus at diagnosis, followed by continuous infusion of 1 g IV every 8 h until treatment of aneurysm or a maximum of 24 h.

g: gram, IV: intravenous, mL: milliliter, N/A: not available, aSAH: aneurysmal subarachnoid hemorrhage, TXA: tranexamic acid. Numbers in parentheses indicate the number of patients receiving medications.

**Table 3:** Comparing the studies’ outcomes between the tranexamic acid groups and the control groups.

Author (Year)	Rebleeding			Mortality Rate			Thromboembolic Events			Cerebral Ischemia			Other Adverse Effects	
	TXA	Control	Total	TXA	Control	Total	TXA	Control	Total	TXA	Control	Total	TXA	Control
Fodstad et al. (1981) [12]	6	7	13	13	9	22	4	3	7	5	2	7	Vasospasm (20), ventricular dilatation (8), MI (2)	Vasospasm (20), ventricular dilatation (12), suspected MI (1)
Fodstad et al. (1982) [13]	7	16	23	13	9	22	8	2	10	7	2	9	Ventricular dilatation (11), MI (2), suspected MI (2)	Ventricular dilatation (15), suspected MI (1)
Roos et al. (2000) [14]	44	77	121	114	105	219	N/A	N/A	N/A	68	74	72	Clinical deteriorations from onset (11), hydrocephalus (71), post-operative ischemia (11), others (41)	Clinical deteriorations from onset (9), hydrocephalus (62), post-operative ischemia (10), others (44)
Hillman et al. (2002) [15]	6	27	33	N/A	120	N/A	0	0	0	17	15	32	N/A	N/A
Post et al. (2021) [10]	42	57	99	128	114	242	37	40	77	108	106	214	N/A	N/A
Tjerkstra et al. (2022) [16]	39	56	95	117	107	224	34	40	74	104	103	207	N/A	N/A

Numbers indicate the incidence of adverse events. MI: myocardial infarction, N/A: not available, TXA: tranexamic acid.

## Data Availability

All data are publicly available.

## Supplementary Materials

The following supporting information can be downloaded at: https://www.mdpi.com/article/10.3390/healthcare12232452/s1, The PRISMA checklist [24].
